# Special Issue “Pharmaceutical Solid Forms: From Crystal Structure to Formulation”

**DOI:** 10.3390/pharmaceutics17030312

**Published:** 2025-02-28

**Authors:** Philippe Espeau

**Affiliations:** Unit of Chemical and Biological Technologies for Health (UTCBS), CNRS, INSERM, Université Paris Cité, 75006 Paris, France; philippe.espeau@u-paris.fr

This Special Issue aims to highlight the interest of characterizing the structural aspects of an API before its formulation, as much work is required between the discovery of a molecule with a therapeutic effect and its formulation. Therapeutic effects depend on the solid state of the API. Indeed, the choice of solid form has an impact on the chemical and physical properties of a drug, particularly on its physical and/or chemical stability (including pharmaceutical operations), dissolution rate, solubility, and bioavailability. In a crystalline state, an anhydrous API can exist in different solid phases. This is called crystalline polymorphism, i.e., the ability of a substance to be able to crystallize in different solid structures.

In general, metastable forms are more soluble than corresponding stable polymorphic forms, but they can transform into a thermodynamically more stable (lower energy) form within a variable time period [1]. For stability reasons, it is always preferable to formulate a drug from its most stable solid form. Relative stability is assessed according to various parameters such as temperature, pressure, water content, pH, etc. However, it may be that, due to insufficient bioavailability, it is necessary to formulate a less stable solid form. It is then necessary to stabilize this metastable form through the right excipients. The physical properties of the active ingredient, as well as its crystal structure, must be characterized using different techniques (X-ray powder diffraction, thermal analyses, etc.) in order to determine which solid form should be formulated.

A new crystalline form of predinosolone (Form 3), obtained via the hydration/dehydration mechanism of a sesquihydrate is reported in this Special Issue (contribution 1).

This crystalline form can also exist in an amorphous state, where the molecules are arranged in a disordered manner. In general, compared with crystalline polymorphs, amorphous forms tend to have a higher dissolution rate and solubility. Compared with crystalline polymorphs, amorphous forms tend to have a higher dissolution rate and solubility, which may increase the rate and extent of their oral absorption.

Two new examples of amorphous pharmaceutical solids are reported in this Special Issue. One is amorphous indomethacin (contribution 2) and one is clotrimazole (contribution 3).

In the presence of another entity, such as co-formers or solvent molecules, such as water, the API can form co-crystals or solvates, respectively. Of course, if the solvent is water, the term hydrate is used. The notion of polymorphism also applies to co-crystals. Numerous examples are reported in the literature [2,3]. In this Special Issue, a new co-crystal similar to ilaprazole and xylitol is reported (contribution 4), and the storage and stability of the co-crystal is investigated. Mixing two API does not necessarily lead to co-crystal formation but may still be of pharmaceutical interest. It is of course appropriate to study the stability of two active ingredients within a drug as well as the possible interactions between them. The compatibility between montelukast and levocetirizine is examined in a different paper in this Special Issue (contribution 5). Both substances are combined to treat respiratory allergic diseases. Montelukast helps to improve asthma symptoms, and levocetirizine is frequently used to treat allergic rhinitis.

Consequently, the identification and control of the solid form of the API in the final drug must be ensured throughout the development program, including its final packaging. Indeed, it may be that the active substance interacts physically or chemically with the excipients or with the container, which could modify its activity.

We must remember the case of ritonavir, which caused wide-spread change in terms of formulation approaches [4]: in the 1990s, several batches of capsules failed their dissolution requirements because a new polymorphic form appeared during the production of a second batch of capsules. This form was an example of conformational polymorphism. It was this case which made the identification of polymorphism obligatory. This example, surely the most “publicized”, is unfortunately not the only case reported in the literature [5]. This search for polymorphs for solid pharmaceutical forms is accompanied, of course, by a study of crystalline structures, among other things, before any possible formulation.
